# The use of personal protective equipment as an independent factor for developing depressive and post-traumatic stress symptoms in the postpartum period

**DOI:** 10.1192/j.eurpsy.2021.29

**Published:** 2021-05-04

**Authors:** Hadar Gluska, Yael Mayer, Noga Shiffman, Rawan Daher, Lior Elyasyan, Nofar Elia, Maya Sharon Weiner, Hadas Miremberg, Michal Kovo, Tal Biron-Shental, Liat Helpman, Rinat Gabbay-Benziv

**Affiliations:** 1 Obstetrics and Gynecology, Meir Medical Center, Kfar Saba, Israel; 2 Sackler Faculty of Medicine, Tel-Aviv University, Tel-Aviv, Israel; 3 Department of Occupational Science and Occupational Therapy, Faculty of Medicine, The University of British Columbia, Vancouver, BC, Canada; 4 Obstetrics and Gynecology, Hillel Yaffe Medical Center, Hadera, Israel; 5 The Ruth and Bruce Rappaport Faculty of Medicine, Haifa, Israel; 6 Obstetrics and Gynecology, Edith Wolfson Medical Center, Holon, Israel; 7 Sackler Faculty of Medicine, Tel-Aviv University, Tel-Aviv, Israel; 8 Department of Counseling and Human Development, Faculty of Education, University of Haifa, Haifa, Israel; 9 Psychiatric Research Unit, Tel Aviv Sourasky Medical Center, Tel-Aviv, Israel

**Keywords:** City birth Trauma Scale (City BiTS), Childbirth, COVID-19, Edinburgh Postnatal Depression Scale (EPDS), personal protective equipment (PPE), postpartum depression (PPD), postpartum post-traumatic stress disorder (PTSD)

## Abstract

**Background:**

New recommendations regarding the use of personal protective equipment (PPE) during delivery have changed the maternal birth experience. In this study, we investigated the mental perceived impact of PPE use during delivery on the development of maternal postpartum depression (PPD) and post-traumatic stress symptoms (PTSS).

**Methods:**

This was a multicenter, retrospective cohort study concerning women who delivered during the COVID-19 pandemic first lockdown period in Israel. Postpartum women were approached and asked to complete a comprehensive online questionnaire. Impact of PPE was graded on a scale of 1–5, and Impact of PPE ≥4 was considered high. PPD and PTSS were assessed using the EPDS and City BiTS questionnaires.

**Results:**

Of 421 parturients, 36 (9%) reported high Impact of PPE. Parturients with high Impact of PPE had significantly higher PPD and PTSS scores)EPDS 8.4 ± 5.8 vs. 5.7 ± 5.3; City BiTS 9.2 ± 10.3 vs. 5.8 ± 7.8, *p <* 0.05 for both). Following adjustment for socio-demographic and delivery confounders and fear of COVID-19 (using Fear of COVID19 scale), Impact of PPE remained positively correlated with PPD symptoms (*ß* = 0.103, 95% confidence intervals [CI] 0.029–1.006, *p* = 0.038).

**Conclusion:**

When examining the risk factors for developing postpartum PTSS—experiences during labor and PPE were found to be significant variables. As the use of PPE is crucial in this era of COVID-19 pandemic in order to protect both parturients and caregivers, creative measures should be taken in order to overcome the communication gap it poses.

## Introduction

In terms of mental health, the COVID-19 pandemic has exerted tremendous influence on the population worldwide, with an increase in the prevalence of anxiety, stress, and depression [1–3]. Pregnant and postpartum women are considered to be an especially vulnerable population, presenting with higher rates of psychopathologies during the pandemic [4–7]. Pregnant women experienced higher levels of fear of COVID-19 infection [8] that was further augmented by fear of endangering the fetus [9]. Additionally, social distancing and restrictive lockdowns enforced by governments, have limited maternal social support networks, as well as access to healthcare services. This in turn has increased maternal risk for the development of psychological disorders [10]. Nevertheless, with emerging data, it is important to note that unlike most studies, some suggest that the maternal change in mood symptomology during the pandemic is not universal and mainly related to maternal socioeconomic status and support [11]. The childbirth experience has also changed dramatically with the new regulations regarding COVID-19 screening, restricted number of partners and staff supporting the parturient, and the use of personal protective equipment (PPE). A cross-sectional online study that evaluated maternal experience with respect to childbirth before and after the pandemic found that “Joy” was the most prevalent emotion expressed before COVID-19 and “Fear” was the most prevalent after. This change was attributed to the fear of the pandemic itself, but also to all protective measures taken during delivery [12].

Postpartum post-traumatic stress disorder (PTSD) stems from the parturiant’s personal experience of childbirth as a stressed, traumatic, frightening, and even life-threatening event [13]. According to the Diagnostic and Statistical Manual of Mental Disorders, fourth edition (DSM-IV) diagnostic criteria for PTSD, this psychopathology is based on intense fear and sense of helplessness. As for postpartum PTSD, these feelings may be attributed to an objective event such as: emergent cesarean delivery, vacuum extraction, life danger to the newborn and more, but also due to maternal sense of powerlessness, loss of control and lack of support, and reassurance during delivery [14].

Support from the maternity staff during childbirth is an essential component that can potentially decrease maternal anxiety and stress [15]. Feelings of lack of control during labor was found to be associated with negative birth experience and adverse maternal mental health outcomes [16]. Face-to-face psychological support, sufficient eye contact, touch, and tone of speech are critical elements of care during any caregiver–patient encounter [17], all of which are interrupted by PPE regulations. Prior to COVID-19 era, there was limited use of PPE by staff during labor: rubber gloves were commonly used, but other elements like goggles were not essential in the setting of labor until the final time of fetal delivery. The lately mandatory continuous use of PPE in COVID-19 times by both the patient and caregivers interferes with the caregiver–patient relationship [18], and may jeopardize the vital midwife-parturient connection, thus, not only fails to support the laboring woman but may further increase her stress and anxiety.

Therefore, in this study, we aimed to investigate the perceived impact of PPE on parturients and to evaluate its association with the development of postpartum post-traumatic stress symptoms (PTSS) and postpartum depressive (PPD) symptoms.

## Methods

This was a secondary analysis of a multicenter, prospective, and observational study that was conducted at three university affiliated medical centers in Israel between March 10, 2020 and May 9, 2020, during the COVID-19 pandemic strict lockdown period. Centers included were Hillel Yaffe Medical Center (HYMC), Meir Medical Center (MMC), and Wolfson Medical Center (WMC). All of which are university affiliated facilities. The study was approved by each medical center’s Institutional Review Board (HYMC-20-0079, MMC-0169-20, WMC-143-20, and NIH NCT04609501). In this study, we aimed to investigate the impact of PPE use by medical staff at birth as a risk factor for the future development of postpartum PTSD and PPD symptoms.

### Study population

Parturients were eligible to participate following live born delivery in one of the three participating medical centers during the above-mentioned period, which was during the first lockdown enforced by the government in Israel to prevent the spread of coronavirus infection. Parturients younger than 18 years or who delivered earlier than 34 gestational weeks were excluded. A comprehensive team of physicians and medical students, both Hebrew and Arabic speaking, approached all women virtually approximately 10 weeks after delivery—the custom time interval from birth that is used to assess PPD and postpartum PTSD symptoms or other birth related psychopathologies [19]. Women were given a brief explanation of the study protocol and were asked to participate in the study. Following oral consent, a text message was sent to each of them, that contained a link to an online questionnaire, either in Hebrew or in Arabic, according to the participant’s preference. Questionnaires were sent using the online Qualtrics survey platform.

### Maternal medical information

Maternal demographics, obstetric history, pregnancy surveillance, labor, and delivery data as well as short term maternal and neonatal outcome (until home discharge) were all retrieved from the comprehensive computerized perinatal database at each medical center. At all centers, data were routinely collected at the time of admission to labor ward, during labor and delivery and at postpartum admission, and was retrospectively retrieved and analyzed. Maternal data included medical background such as age, pre-gestational weight and height, any known cardiovascular or metabolic illness, medications including any psychiatric medications, smoking, and so on. We used psychiatric medication as a surrogate variable for any previous maternal mental health disorder or symptomology as in our experience, both women and physicians do not always report mild psychological disorders on admission, however, accurately report on medication for neonatal safety. Obstetric characteristics included parity, previous obstetric history, and current pregnancy follow up (first and second trimester genetic screening, anatomy scan, glucose status, and any hypertensive disorders). Parameters regarding the course of labor were also included—gestational age at delivery, need for induction of labor, anesthesia, mode of delivery, and any birth complications (fever during/after labor, post-partum hemorrhage, obstetric anal sphincter injury, postdelivery operations, etc). Birth outcomes included newborn’s weight, Apgar scores and umbilical cord blood pH. In addition, variables concerning the postpartum course of both the mother and the newborn were collected: the number of hospitalization days, admission to a maternal or neonatal intensive care unit, breastfeeding, and more.

### Online questionnaires

Using the Qualtrics survey platform, women were asked to provide demographic, socioeconomic, and obstetrical information, and to complete an assembly of mental health questionnaires.

Demographic and socioeconomic details included questions regarding ethnicity, religious tendencies, education, family status, work status, average household income on a 5-point scale defined in relation to the average household income in Israel (1—“Far below average”, 5—“Far above average”).

Two specific COVID-19 pandemic questionnaires were included: first regarding PPE that included two questions: (a) whether the medical staff at birth used PPE (by maternal recollection), and if so, which equipment was in use (facemasks, rubber gloves, protective goggles or shield, disposable gown, or none of the mentioned) and (b) the extent to which participants experienced difficulty as a result, on a 5-point scale (1—“not difficult at all”; 2—“a little bit difficult”; 3—” fairly difficult”; 4—” very difficult”; and 5—“extremely difficult”). We named this parameter “Impact of PPE” and evaluated it both as continuous and categorical values with score of 1–3 considered low Impact of PPE and score of 4–5 considered high Impact of PPE. This categorization was not chosen arbitrarily, it was guided by the scale, as a score of 4 or 5 refers to extensive negative impact of PPE use.

“Fear of COVID-19 Scale” was the second questionnaire. It is a novel validated questionnaire that was designed to assess different aspects of the fear of the pandemic, and was found to be associated with anxiety, stress, and depression in the general population [20]. To note, this questionnaire was also validated for the Hebrew language [21]. The questionnaire includes seven statements such as “I am afraid of losing my life because of the Coronavirus.” Participants were asked to rate their degree of agreement with the statements on a 5-point scale (total score 7–35).

To evaluate stress and anxiety that may have originated from objective potential events during pregnancy and delivery, we further defined two variables: *Stress-contributing complications during pregnancy* that reflect the need for a high-risk clinical follow-up during pregnancy. These that were defined as the presence of one of the following: gestational diabetes, any hypertensive disorder, fetal growth restriction or major risk of prematurity defined as need for cerclage. The second variable, *Stress-contributing complications during delivery* that reflect unexpected negative outcomes during delivery and include one of the following: need for an urgent cesarean delivery, explorative relaparotomy or unplanned hysterectomy, need for a blood transfusion, any anal sphincter injury or need for admission to maternal or neonatal intensive care units.

PTSD diagnosis and symptoms were evaluated using the validated City Birth Trauma Scale )City BiTS(, a self-report questionnaire aimed at assessing PTSD following childbirth, based on the DSM-5 criteria [22] that was translated to Hebrew [23]. The questionnaire consists of 29 items dealing with possible traumatic events during or immediately after birth, of them, 22 items refers to symptomology. The questionnaire is divided into diagnostic criteria, as follows: A—stressor criteria, B—re-experiencing symptoms, C—avoidance symptoms, D—negative cognition and mood, and E—hyperarousal. Participants are asked to respond on a 4-point scale (not at all, once, 2–4 times and 5 or more times). Total PTSD score was calculated as the sum of criteria B–E (total score of 22 questions). PTSD was evaluated as a continuous variable for each criterion separately and for total PTSD symptoms. Also, PTSD diagnosis was determined if participant replied any answer other than “not at all” to at least one question in criteria A–C and at least two questions for criteria D–E. PPD diagnosis and symptoms were evaluated using the validated Edinburgh Postnatal Depression Scale )EPDS(questionnaire [24]. This questionnaire is composed of 10 items, scored by using a 4-point scale (0–3). A score of ≥10 was considered as possible depression diagnosis [24–26] and is commonly used in Israel as a threshold that requires further evaluation for detection of PPD symptoms. Depression was evaluated as a continuous variable to evaluate PPD symptoms [27]. Ultimately, all collected data were verified with each center’s perinatal and postnatal database.

For the Arabic version, previously translated and validated questionnaires were used, or alternatively, questionnaires were translated and back-translated by native Arabic speakers. A complete set of questionnaires is available as a Supplementary Material.

### Statistical analysis

The final analysis included only women who answered at least 70% of the questionnaire. Data analysis was performed with the SPSS v23.0 package (IBM Corp., Chicago, IL).

Univariate analysis was used to stratify maternal demographics, delivery, and postnatal data with low or high Impact of PPE (≥4 high Impact of PPE). Continuous variables were compared using Mann–Whitney *U* test and categorical variables were compared using the chi-square or Fisher’s exact tests, as appropriate.

Correlations between Impact of PPE scores and EPDS/City BiTS total scores were evaluated using the Pearson correlation test. Correlations between each PTSD criteria, defined by the City BiTS questionnaire as a categorial variable, were evaluated using the Spearman correlation test.

Positive correlations were further analyzed using a three-stage forward stepwise linear regression analysis to adjust outcomes for potential confounders. Stage 1 included maternal demographic variables and potential confounders related to predelivery maternal health: maternal age, ethnicity, family status, education, average income, and predelivery maternal mental health disorder. Stage 2 added variables related to birth experience and included *Stress-contributing complications during delivery* and Impact of PPE. Stage 3 was added to include Fear of COVID-19 as a confounder that may increase maternal PPD and PTSD symptoms. Differences were considered significant when *p*-value was less than 0.05.

## Results

Overall, 1,462 women delivered during the duration of the study at the three participating medical centers. Of them, 1,079 (74%) were approached by phone and asked to be included in the study. In total, 774 (53%) consented to answering the online questionnaires and 421 (29%) answered over 70% of the questionnaires, and thus entered the analysis. Mean delivery to questionnaire interval was 11.0 ± 1.6 weeks.

For the entire cohort, mean maternal age was 31.5 ± 5.3 years. Of them, 78.1% were Jewish and 22.9% were Arabic. Overall, 96.1% of the participants were married or in a relationship and for 71.7% this was their first delivery. The mean gestational age at delivery was 39.5 ± 1.2 weeks and the mean neonatal birthweight was 3271 ± 407 g. Three hundred and forty-two women (81.2%) delivered vaginally and the rest (79, 18.8%) by a cesarean section. Of the 79 cesarean deliveries, 41 were urgent.

Mean EPDS score for the entire cohort was 6 ± 5.4. Ninety women (23%) had high EPDS scores (≥10 points). Mean City BiTS total score was 6.1 ± 8.1 and 14 women (3%) fulfilled the PTSD diagnosis (at least one question answered “yes” in criteria A–C and at least two questions answered “yes” in criteria D–E).

As for the type of PPE used at birth by medical staff, 380 (90.3%) women reported that facemasks were in use, 296 (70.3%) reported rubber gloves, 175 (41.6%) reported disposable gown, 45 (10.7%) reported the use of protective goggles or shields, and 29 (6.9%) stated that no PPE was in use. Impact of PPE was reported by 391/421 (92.9%) women. On a 1–5 scale (1—“not difficult at all”; 2—“a little bit difficult”; 3—“fairly difficult”; 4—“very difficult”; and 5—“extremely difficult”), mean Impact of PPE was 1.8 ± 1.1. High Impact of PPE, defined as Impact of PPE ≥ 4, was experienced by 36/391 (9.2%) of the participants. Demographic, socioeconomic, and obstetric data of the study cohort stratified by Impact of PPE score are presented in Tables 1 and 2. No differences were found between the groups in terms of age, parity, ethnicity, education, marital status, and religious level or psychiatric background (deduced by report of psychiatric drugs).

**Table 1.** Maternal characteristics by Impact of PPE score.

	Impact of PPE LOW (1–3) *N* = 352	Impact of PPE HIGH (4–5) *N* = 36	*p*
Impact of PPE scale	1.6 ± 0.8	4.4 ± 0.5	**0.000**
Maternal age	31.5 ± 5.2	30.8 ± 5.5	0.323
Nulliparity	100 (28.2%)	12 (33.3%)	0.317
Chronic hypertension	8 (2.3%)	1 (2.8%)	0.585
Pregestational diabetes	4 (1.1%)	1 (2.8%)	0.385
Maternal disability^a^	30 (8.5%)	4 (11.1%)	0.384
Smoking	16 (4.5%)	4 (11.1%)	0.101
Alcohol	0	0	–
Drugs	0	0	–
Psychiatric drugs	5 (1.4%)	2(5.6%)	0.129
Ethnicity:			
Jews	278 (78.3%)	28 (77.8%)	
Arabic	77 (21.7%)	8 (22.2%)	0.542
Marital Status			
Married	321 (90.4%)	32 (88.9%)	
In relationship	23 (6.5%)	1 (2.8%)	
Separated/single	11 (3.1%)	3 (8.3%)	0.198
Religious level (scale 0–4; 0—secular to 4—very religious)	1.8 ± 0.8	1.7 ± 0.7	0.789
Education:			
Elementary	5 (1.4%)	0 (0.0%)	
High school	127 (35.8%)	21 (58.3%)	
Bachelor’s degree	155 (43.7%)	10 (27.8%)	
Master’s degree	61 (17.2%)	5 (13.9%)	
Doctorate	7 (2.0%)	0 (0.0%)	0.1
Average household income			
Far below average	63 (17.7%)	8 (22.2%)	
Below average	95 (26.8%)	10 (27.8%)	
Average	106 (29.9%)	9 (25.0%)	
Above average	77 (21.7%)	9 (25.0%)	
Far above average	14 (3.9%)	0 (0.0%)	0.696

For categorical variables results are presented as *n* (%) and for continuous variables as mean ± standard deviation (SD). Significant *p* values (<0.05) are in bold.

Abbreviation: PPE, personal protective equipment.

a Maternal disability—any prior physiological or psychological chronic health condition (per maternal view).

**Table 2.** Characteristics and outcome by Impact of PPE score.

	Impact of PPE LOW (1–3)	Impact of PPE HIGH (4–5)	*P*
Gestational week at delivery	39.5 ± 1.1	39.28 ± 1.7	0.798
Birth weight (g)	3273.3 ± 400.8	3223.5 ± 521.1	0.556
Fertility treatment	26 (7.7%)	2 (6.1%)	0.536
Multiple pregnancy	1 (0.3%)	0 (0.0%)	0.908
Male gender	147 (53.1%)	17 (73.9%)	**0.041**
Gestational diabetes^a^	22 (6.2%)	2 (5.6%)	0.245
Hypertensive disorder^b^	20 (5.6%)	2 (5.6%)	0.361
Induction of labor	112 (38.0%)	16 (47.1%)	0.199
Mode of delivery			
Vaginal delivery	261 (73.5%)	24 (66.7%)	
Vacuum delivery	28 (7.9%)	5 (13.9%)	
Elective cesarean	32 (9.0%)	3 (8.3%)	
Urgent cesarean	34 (9.6%)	4 (11.1%)	0.637
Maternal ICU admission	4 (1.1%)	2 (5.6%)	0.098
Neonatal ICU admission (term)	12 (3.4%)	2 (5.9%)	0.353
OASIS	1 (0.3%)	0 (0.0%)	0.908
Blood transfusion	6 (1.7%)	3 (8.3%)	**0.041**
Stress-contributing complications during pregnancy^c^	45 (12.7%)	5 (13.9%)	0.5
Stress contributing complications during delivery^d^	55 (15.5%)	7 (19.4%)	0.338
Maternal hospitalization length (days)	3.4 ± 1.1	3.3 ± 0.9	0.684
Neonatal hospitalization length (days)	3.1 ± 1.36	5.5 ± 15.2	0.674
Breastfeeding	267 (78.8%)	25 (71.4%)	0.213
Time interval for questionnaire (weeks)	11.1 ± 1.7	10.7 ± 1.3	0.167
FEAR OF COVID 19 scale^e^	17.5 ± 5.4	18.9 ± 6.7	0.295

For categorical variables results are presented as value (%) and for continuous variables as value ± standard deviation (SD). Significant *p* values (<0.05) are in bold.

Abbreviations: ICU, intensive care unit; OASIS, obstetrical anal sphincter injury; PPE, personal protective equipment.

a Gestational diabetes—including gestational diabetes mellitus with diet treatment (GDMA1) and pharmacological treatment (GDMA2)

b Hypertensive disorder—including gestational hypertension and preeclampsia.

c Stress-contributing complications during pregnancy including any gestational diabetes, hypertensive disorder, fetal growth restriction or major risk of prematurity defined as need for cerclage.

d Stress-contributing complications during delivery including any need for urgent cesarean delivery, relaparotomy or unplanned hysterectomy, need for blood transfusion, any anal sphincter injury or need for maternal or neonatal intensive care unit admission.

e Fear of COVID 19—evaluated by the Fear of COVID-19 scale [20].

Compared to postpartum women who reported low Impact of PPE, postpartum women who reported high Impact of PPE had significantly higher EPDS scores (8.4 ± 5.8 vs. 5.7 ± 5.3, *p* = 0.005) and total City BiTS scores (9.2 ± 10.3 vs. 5.8 ± 7.8, *p* = 0.014; Table 3). This association remained significant when Impact of PPE was evaluated as a continuous variable using Pearson correlation (*r* = 0.150, *p* = 0.003 and *r* = 0.109, *p* = 0.035, for EPDS and total City BiTS scores, respectively). As for specific PTSD criteria, represented by the City BiTS A–E criteria, women that reported high Impact of PPE had higher rates of fulfilling criteria B (re-experiencing symptoms) and criteria C (avoidance symptoms) (B criteria: 1.6 ± 13 vs. 2.8 ± 14, *p* = 0.032 and C criteria 0.3 ± 6 vs. 1.2 ± 6, *p* = 0.000). Spearman correlation, evaluating Impact of PPE as a continuous variable, demonstrated similar findings (*r* = 0.112, *p* = 0.032 and *r* = 0.208, *p* = 0.000, for B and C criteria, respectively).

**Table 3.** EPDS and total City BiTS scores stratified by Impact of PPE.

	Impact of PPE LOW (1–3)	Impact of PPE HIGH (4–5)	*p*
EPDS score	5.7 ± 5.3	8.4 ± 5.8	**0.005**
City BiTS total score	5.8 ± 7.8	9.2 ± 10.3	**0.014**
PTDS criterion A	0.2 ± 0.5	0.2 ± 0.4	0.869
PTDS criterion B	1.6 ± 2.5	2.8 ± 3.6	**0.032**
PTDS criterion C	0.3 ± 0.9	1.2 ± 1.8	**0.000**
PTDS criterion C	2.0 ± 3.1	2.6 ± 3.4	0.178
PTDS criterion E	2.2 ± 3.3	3.2 ± 4.7	0.379
PTSD diagnosis	11(3.3%)	2(6.1%)	0.327

EPSD was evaluated as continuous scale [24]; PTSD was evaluated as total City BiTS score, while PTSD diagnosis is defined when patient replied “yes” on at least one question in criteria A–C and on at least two questions on criteria D and E [22].

Abbreviations: EPDS, Edinburgh Postnatal Depression Scale; PPE, personal protective equipment; PTSD, post-traumatic stress disorder.

Lastly, in order to adjust the relation between Impact of PPE and postpartum PTSD and PPD symptoms to other confounders, we performed logistic regression analysis in three stages, referring to their socio-demographic characteristics at the first stage, birth experience including the *Stress-contributing complications during delivery* and Impact of PPE at the second stage, and at the last stage adding the pandemic stressor manifested by the Fear of COVID-19 variable, utilizing the “Fear of COVID-19 Scale” questionnaire [20] (shown in Tables 4 and 5). At the first stage, higher EPDS scores were associated with higher education and lower household income (Beta = 0.137, 95% CI 0.198–1.171, *p* = 0.013 and Beta = −0.136, 95% CI −1.215 to −0.116, *p* = 0.018, respectively). Following addition of variables associated with birth experience (Impact of PPE and *Stress-contributing complications during delivery*) higher Impact of PPE was found to be a significant variable (Beta = 0.161, 95% CI 0.312–1.317, *p* = 0.002) positively affecting EPDS score. Last, even after addition of Fear of COVID-19 at the last stage of the analysis, the variables that remained significant predictor of higher EPDS scores were level of education, Impact of PPE and Fear of COVID-19 (Beta = 0.151, 95% CI 0.328–1.759, *p* = 0.004; Beta = 0.103, 95% CI 0.029–1.006, *p* = 0.038; Beta = 0.309, 95% CI 0.207–0.400, *p* = 0.000, respectively).

**Table 4.** Three-stage statistical linear regression analysis for EPDS scores.

	Beta	95% confidence interval		*p* value	*R* ^2^
		Lower bound	Upper bound		
First stage					
Age	−0.084	−0.203	0.029	0.142	
Ethnicity^a^	−0.035	−1.775	0.846	0.486	
Marital status^b^	0.061	−0.602	2.119	0.274	
Education^c^	0.137	0.198	1.706	**0.013**	
Average household income	−0.136	−1.215	−0.116	**0.018**	
Psychiatric medications	−0.020	−4.869	3.271	0.700	0.034
Second stage					
Age	−0.075	−0.193	0.037	0.182	
Ethnicity^a^	−0.035	−1.762	0.848	0.491	
Marital status^b^	0.063	−0.564	2.127	0.254	
Education^c^	0.157	0.339	1.839	**0.005**	
Average household income^d^	−0.139	−1.224	−0.137	**0.014**	
Psychiatric medications	−0.027	−5.140	2.923	0.589	
Stress-contributing complications during delivery^e^	0.019	−1.181	1.756	0.701	
Impact of PPE	0.161	0.312	1.317	**0.002**	0.060
Third stage					
Age	−0.053	−0.165	0.055	0.324	
Ethnicity^a^	−0.034	−1.692	0.796	0.479	
Marital status^b^	0.093	−0.131	2.446	**0.078**	
Education^c^	0.151	0.328	1.759	**0.004**	
Average household income^d^	−0.098	−1.001	0.043	**0.072**	
Psychiatric medications	−0.008	−4.177	3.527	0.868	
Stress-contributing complications during delivery^e^	0.013	−1.205	1.595	0.784	
Impact of PPE	0.103	0.029	1.006	**0.038**	
Fear of COVID-19^f^	0.309	0.207	0.400	**0.000**	0.148

Abbreviations: EPDS, Edinburgh Postnatal Depression Scale; PPE, personal protective equipment.

a Ethnicity*—*Jews and Arabic.

b Marrital status—married, in relationship, separated, and single.

c Education—elementry, high school, bachelor’s degree, master’s degree, doctorate.

d Average household income—on a 5-point scale (defined in relation to the average household income in Israel (from 1—“far below average” to 5—“far above average”).

e Stress-contributing complications during delivery including any need for urgent cesarean delivery, relaparotomy or unplanned hysterectomy, need for blood transfusion, any anal sphincter injury or need for maternal or neonatal intensive care unit admission.

f Fear of COVID 19—evaluated by the Fear of COVID-19 scale [20].

**Table 5.** Three-stage statistical linear regression analysis for City BiTS scores.

	Beta	95% confidence interval		*p* value	*R* ^2^
		Lower bound	Upper bound		
First stage					
Age	−0.060	−0.268	0.083	0.299	
Ethnicity^a^	−0.063	−3.203	0.745	0.222	
Marital status^b^	0.032	−1.474	2.678	0.569	
Education^c^	0.111	0.002	2.275	**0.050**	
Average household income	−0.053	−1.219	0.441	0.357	
Psychiatric medications	0.120	1.114	13.286	**0.021**	0.032
Second stage				0.218	
Age	−0.056	−0.260	0.087	0.326	
Ethnicity^a^	−0.050	−2.933	0.997	0.333	
Marital status^c^	0.032	−1.453	2.650	0.567	
Education^d^	0.125	0.155	2.412	**0.026**	
Average household income^e^	−0.050	−1.189	0.451	0.377	
Psychiatric medications	0.111	0.638	12.686	**0.030**	
Stress-contributing complications during delivery^f^	0.126	0.555	4.952	**0.014**	
Impact of PPE	0.109	0.063	1.581	**0.034**	0.060
Third stage				0.471	
Age	−0.043	−0.238	0.103	0.439	
Ethnicity^a^	−0.048	−2.868	0.988	0.338	
Marital status^b^	0.049	−1.088	2.953	0.365	
Education^c^	0.123	0.151	2.366	**0.026**	
Average household income^d^	−0.026	−1.001	0.618	0.642	
Psychiatric medications	0.124	1.511	13.362	**0.014**	
Stress-contributing complications during delivery^e^	0.121	0.496	4.813	**0.016**	
Impact of PPE	0.069	−.240	1.281	0.179	
Fear of COVID-19^f^	0.200	0.146	0.449	**0.000**	0.097

Abbreviations: PPE, personal protective equipment.

a Ethnicity*—*Jews, Arabic.

b Marrital status—married, in relationship, separated, and single.

c Education—elementry, high school, bachelor’s degree, master’s degree, and doctorate.

d Average household income—on a 5-point scale (defined in relation to the average household income in Israel (from 1—“far below average” to 5—“far above average”).

e Stress-contributing complications during delivery including any need for urgent cesarean delivery, relaparotomy or unplanned hysterectomy, need for blood transfusion, any anal sphincter injury or need for maternal or neonatal intensive care unit admission.

f Fear of COVID 19—evaluated by the Fear of COVID-19 scale [20].

As for PTSS, represented by the City BiTS total scores, in the first stage, higher levels of education and the use of psychiatric medications were associated with higher risk for developing PTSS (Beta = 0.111, 95% CI 0.002–2.275, *p* = 0.050; Beta = 0.120, 95% CI 1.114–13.286, *p* = 0.021, respectively). In the second stage, Impact of PPE and *Stress-contributing complications during delivery* were both found to be significant variables (Beta = 0.109, 95% CI 0.063–1.581, *p* = 0.034; Beta = 0.126, 95% CI 0.555–4.952, *p* = 0.014, respectively). When adding Fear of COVID-19 at the last stage, Impact of PPE was no longer a predictor.

## Discussion

In this study, we aimed to investigate the impact of PPE use during childbirth and to evaluate its association with postpartum PPD and PTSD symptoms, during the COVID-19 pandemic. In our cohort, 36 women, which represent 9.2% of the study population, reported high Impact of PPE. In addition, 23.5% of the participants presented PPD symptoms (EPDS score ≥ 10 points) and 3% met all the criteria for diagnosis of postpartum PTSD. The main finding of our study was that women who reported healthcare providers’ use of PPE as difficult, that is women with higher Impact of PPE, experienced higher levels of PPD and postpartum PTSS, compared to their counterparts. For PPD, this remained true after adjusting symptoms to stressors originating from maternal demographics, medical history, delivery characteristics, and the fear of COVID-19.

During any medical encounter, patient–caregiver communication is composed of at least 55% nonverbal communication [28], including facial expressions, eye contact, and manner of speech [29]. This is especially important in the setting of childbirth, which is a unique, emotional, and intimate event, in which there is great value to the caregiver–parturient relationship [30,31]. The use of facemasks by healthcare providers was found to negatively influence the level of empathy, thus interfering with caregiver–patient relationship [32,33]. A recent study has found that the use of PPE by medical staff during childbirth created communication barriers between caregivers and parturients [4].

Previous studies investigated the risk factors associated with an increased risk of PPD and PTSD symptoms. Both the use of PPE and fear of COVID-19 may further increase this risk. Our study demonstrated that even after adjusting outcomes to fear of COVID-19, Impact of PPE remained a significant variable that contributed to maternal PPD symptoms. Interestingly and unlike PPD symptoms, we discovered that although Impact of PPE and *stress-contributing complications during delivery* were associated with increased PTSD symptoms, when we added the fear of COVID-19 as a variable to the regression, Impact of PPE was no longer a significant factor. This difference originates from the fundamental discrepancy between PPD and PTSD. While both types of symptoms may be related to the experience during birth, as found in pre-COVID-19 studies, PPD development was found to be more related specifically to caregiver–parturient relationship [34]. However, postpartum PTSD was associated with factors such as feelings of fear or lack of control during childbirth [35,36], and to a negative birth experience not as an absolute, but in comparison to the expected [37].

To the best of our knowledge, this is the first study to examine the effects of PPE on birth experience and mental well-being of women who gave birth during the current pandemic. Our study benefits from being a multicenter cohort study, which allowed us to reach a diverse population in terms of ethnicity, religion, and socioeconomic status. Exclusion of women who delivered before 34 gestational weeks, minimized the bias of the development of psychopathology symptoms due to prematurity [38]. Unlike other studies [39-41], we did not use social media to recruit patients, thus decreasing the bias associated with accessibility to technology or involvement with social networks. In addition, at least some of the maternal reports on demographics, obstetric and delivery data were validated with their medical records. Lastly, we specifically approached women about 10 weeks after labor, which is the acceptable time interval for detection of postpartum related psychopathologies.

Nevertheless, our study is not free of limitations. A substantial number of women did not answer the recruitment phone call, which reflects a response bias, but one perhaps less significant compared to approaching participants through social media. Also, we reported selection bias as not all women who consented actually responded to 70% of the questionnaires. There may be several considerable reasons that may cause women not to fulfill the questionnaire after recruitment, thus may skew the results in all sorts of directions. It can be assumed, for example, that women who did not answer the questionnaire in full represent a more depressed or mentally ill subpopulation, whose mental state did not allow them to touch on such sensitive issues during this time period. On the other hand, we cannot rule out that women who did not answer the questionnaire were those with higher mental strengths, who did not feel the need, and did not understand the importance of participating in the study. The truth probably lies somewhere in between. Concerning our main variable, impact of PPE, one may consider this as only a partial evaluation as we based our maternal perception of difficulty using only one question. However, as the current pandemic confronted us for the first time with a new standard of widespread PPE use and new communication challenges on an extensive scale, there is not yet a validated questionnaire for the assessment of the use of PPE, and its mental impact on patients, and in particular on parturients. Lastly, our study is limited by its cross-sectional nature and causal interpretations should be made with caution. In light of this, further studies should focus on evaluating how PPE use affects the caregiver–patient relationship.

Surprisingly, not all women reported similar PPE use and 6.9% of participants reported a total lack of PPE use during their delivery. This contradicts the strict regulations that were enforced in all delivery centers in Israel during the study period. It may be assumed that this refers to maternal recollection of birth and not to the actual use of PPE by medical staff. Even so, the variable in question was not the use of PPE but the subjective difficulty of the parturient. Lastly, we should acknowledge that there may be inaccuracies in the patients’ descriptions of the impact of the birth experience on their feelings, as we base our results on online questionnaires without in-person psychological evaluation of the parturients. Further qualitative research could broaden our knowledge and present a more complete picture regarding parturients’ birth experiences during the COVID-19 pandemic.

It is clear that PPE is of utmost importance to protect medical staff and the parturient. Nonetheless, we should be aware of the difficulties it poses on the caregiver–parturient relationship and the potential elevation of risk for developing postpartum PTSD or PPD symptoms. These postpartum psychopathologies may cause long-term complications for both the mother and the newborn [42,43]. It is conceivable that once healthcare professionals are vaccinated, it would be possible to alleviate some use of PPE and to create a better birth experience. In the meanwhile, this study stresses the important implications for medical teams and mental health providers. First, creative measures are needed in order to overcome the gaps following the use of PPE in delivery suites in order to strengthen the sense of communication and security among parturients. In addition, in light of the results of this study, parturients could be given information prior to their deliveries, on the PPE that is going to be used by the team, ways to increase communication with the team in this situation, and encouragement to processes their feelings about this unique birth experience. In any case, since PPE use was found in this study to be an important factor related to PPD and PTSS, healthcare providers and medical teams should collect information about PPE use at birth and consider it as a potential risk factor for psychological distress among parturients, and provide the opportunity for women to process the feelings and experiences associated with PPE use.

## Data Availability

The data that support the findings of this study are available on request from the corresponding author, H.G. The data are not publicly available due to restrictions, for example, their containing information that could compromise the privacy of research participants.

## Supplementary Materials

To view supplementary material for this article, please visit http://dx.doi.org/10.1192/j.eurpsy.2021.29. click here to view supplementary material

## References

[1] Kontoangelos K , Economou M , Papageorgiou C . Mental health effects of COVID-19 pandemia: a review of clinical and psychological traits. Psychiatry Investig. 2020;17(6):491. 10.30773/pi.2020.0161 PMC7324731 32570296

[2] Pfefferbaum B , North CS . Mental health and the Covid-19 pandemic. N Engl J Med. 2020;383(6):510–2. 32283003 10.1056/NEJMp2008017

[3] Hamel L , Kearney A , Kirzinger A , Lopes L , Muñana C , Brodie M. KFF health tracking poll–June, https://www.kff.org/report-section/kff-health-tracking-poll-june-2020-social-distancing-delayed-health-care-and-a-look-ahead-to-the-2020-election/; 2020.

[4] Karavadra B , Stockl A , Prosser-Snelling E , Simpson P , Morris E . Women’s perceptions of COVID-19 and their healthcare experiences: a qualitative thematic analysis of a national survey of pregnant women in the United Kingdom. BMC Pregnancy Childbirth. 2020;20(1):1–8. 10.1186/s12884-020-03283-2 PMC7539281 33028237

[5] Saccone G , Florio A , Aiello F , Venturella R , De Angelis MC , Locci M , et al. Psychological impact of coronavirus disease 2019 in pregnant women. Am J Obstetr Gynecol. 2020;223(2):293–5. 10.1016/j.ajog.2020.05.003 PMC7204688 32387321

[6] Preis H , Mahaffey B , Heiselman C , Lobel M . Pandemic-related pregnancy stress and anxiety among women pregnant during the coronavirus disease 2019 pandemic. Am J Obstetr Gynecol MFM. 2020;2(3):100155. 10.1016/j.ajogmf.2020.100155 PMC7295479 32838261

[7] Mappa I , Distefano FA , Rizzo G . Effects of coronavirus 19 pandemic on maternal anxiety during pregnancy: a prospectic observational study. J Perinat Med. 2020;48(6):545–50. 32598320 10.1515/jpm-2020-0182

[8] Salehi L , Rahimzadeh M , Molaei E , Zaheri H , Esmaelzadeh-Saeieh S . The relationship among fear and anxiety of COVID-19, pregnancy experience, and mental health disorder in pregnant women: a structural equation model. Brain Behav. 2020;10(11):e01835. 32969190 10.1002/brb3.1835 PMC7536966

[9] Haruna M , Nishi D . Perinatal mental health and COVID-19 in Japan. Psychiatry Clin Neurosci. 2020;74(9):502–3. 32579265 10.1111/pcn.13091 PMC7362146

[10] Coxon K , Turienzo CF , Kweekel L , Goodarzi B , Brigante L , Simon A , et al. The impact of the coronavirus (COVID-19) pandemic on maternity care in Europe. Midwifery. 2020;88:102779. 32600862 10.1016/j.midw.2020.102779 PMC7286236

[11] Silverman ME , Burgos L , Rodriguez ZI , Afzal O , Kalishman A , Callipari F , et al. Postpartum mood among universally screened high and low socioeconomic status patients during COVID-19 social restrictions in new York City. Sci Rep. 2020;10(1):1–7. 33361797 10.1038/s41598-020-79564-9 PMC7759569

[12] Ravaldi C , Wilson A , Ricca V , Homer C , Vannacci A . Pregnant women voice their concerns and birth expectations during the COVID-19 pandemic in Italy. Women Birth. 2020;S1871–5192(20):30280–8. 10.1016/j.wombi.2020.07.002 PMC7357495 32684343

[13] Polachek IS , Harari LH , Baum M , Strous RD . Postpartum post-traumatic stress disorder symptoms: the uninvited birth companion. Isr Med Assoc J. 2012;14(6):347–53. 22891394

[14] Creedy DK , Shochet IM , Horsfall J . Childbirth and the development of acute trauma symptoms: incidence and contributing factors. Birth. 2000;27(2):104–11. 11251488 10.1046/j.1523-536x.2000.00104.x

[15] Ayers S . Fear of childbirth, postnatal post-traumatic stress disorder and midwifery care. Midwifery. 2014;30(2):145–8. 24365717 10.1016/j.midw.2013.12.001

[16] Green JM , Coupland VA , Kitzinger JV . Expectations, experiences, and psychological outcomes of childbirth: a prospective study of 825 women. Birth. 1990;17(1):15–24. 2346576 10.1111/j.1523-536x.1990.tb00004.x

[17] Larsen KM , Smith CK . Assessment of nonverbal communication in the patient–physician interview. J Fam Pract. 1981;12(3):481–8. 7462949

[18] Marler H , Ditton A . “I’m smiling back at you”: exploring the impact of mask wearing on communication in healthcare. Int J Lang Commun Disord. 2020;56(1):205–14. 33038046 10.1111/1460-6984.12578 PMC7675237

[19] Moraes GP , Lorenzo L , Pontes GA , Montenegro MC , Cantilino A . Screening and diagnosing postpartum depression: when and how? Trends Psychiatry Psychother. 2017;39(1):54–61. 28403324 10.1590/2237-6089-2016-0034

[20] Ahorsu DK , Lin CY , Imani V , Saffari M , Griffiths MD , Pakpour AH . The fear of COVID-19 scale: development and initial validation. Int J Ment Heal Addict. 2020;27:1–9. 10.1007/s11469-020-00270-8 PMC7100496 32226353

[21] Bitan DT , Grossman-Giron A , Bloch Y , Mayer Y , Shiffman N , Mendlovic S . Fear of COVID-19 scale: psychometric characteristics, reliability and validity in the Israeli population. Psychiatry Res. 2020;289:113100. 10.1016/j.psychres.2020.113100 PMC7227556 32425276

[22] Ayers S , Wright DB , Thornton A . Development of a measure of postpartum PTSD: the city birth trauma scale. Front Psych. 2018;9:409. 10.3389/fpsyt.2018.00409 PMC6153962 30279664

[23] Handelzalts JE , Hairston IS , Matatyahu A . Construct validity and psychometric properties of the Hebrew version of the City birth trauma scale. Front Psychol. 2018;9:1726. 30279671 10.3389/fpsyg.2018.01726 PMC6153334

[24] Cox JL , Holden JM , Sagovsky R . Detection of postnatal depression: development of the 10-item Edinburgh postnatal depression scale. Br J Psychiatry. 1987;150(6):782–6. 3651732 10.1192/bjp.150.6.782

[25] Schaper AM , Rooney BL , Kay NR , Silva PD . Use of the Edinburgh postnatal depression scale to identify postpartum depression in a clinical setting. J Reprod Med. 1994;39(8):620–4. 7996526

[26] Gibson J , McKenzie-McHarg K , Shakespeare J , Price J , Gray R . A systematic review of studies validating the Edinburgh postnatal depression scale in antepartum and postpartum women. Acta Psychiatr Scand. 2009;119(5):350–64. 19298573 10.1111/j.1600-0447.2009.01363.x

[27] Cox J , Holden J . Perinatal mental health: a guide to the Edinburgh postnatal depression scale (EPDS). London, UK: Royal College of Psychiatrists; 2003.

[28] Kusnanto H . Patient-centered care. Rev Primary Care Pract Edu. 2018;1(2):51–2.

[29] Pamungkasih W , Sutomo AH , Agusno M . Description of patient acceptance of use of mask by doctor at poly out-patient care Puskesmas. Bantul Rev Primary Care Pract Edu. 2019;2(2):70–5.

[30] Karlström A , Nystedt A , Hildingsson I . The meaning of a very positive birth experience: focus groups discussions with women. BMC Pregnancy Childbirth. 2015;15(1):1–8. 26453022 10.1186/s12884-015-0683-0 PMC4600272

[31] Dahlberg U , Aune I . The woman’s birth experience—the effect of interpersonal relationships and continuity of care. Midwifery. 2013;29(4):407–15. 23399319 10.1016/j.midw.2012.09.006

[32] Forgie SE , Reitsma J , Spady D , Wright B , Stobart K . The “fear factor” for surgical masks and face shields, as perceived by children and their parents. Pediatrics. 2009;124(4):e777–81. 19786438 10.1542/peds.2008-3709

[33] Wong CK , Yip BH , Mercer S , Griffiths S , Kung K , Wong MC , et al. Effect of facemasks on empathy and relational continuity: a randomised controlled trial in primary care. BMC Fam Pract. 2013;14(1):1–7. 24364989 10.1186/1471-2296-14-200 PMC3879648

[34] Bell AF , Andersson E . The birth experience and women’s postnatal depression: a systematic review. Midwifery. 2016;39:112–23. 27321728 10.1016/j.midw.2016.04.014

[35] Polachek IS , Dulitzky M , Margolis-Dorfman L , Simchen MJ . A simple model for prediction postpartum PTSD in high-risk pregnancies. Arch Womens Ment Health. 2016;19(3):483–90. 26399873 10.1007/s00737-015-0582-4

[36] Polachek IS , Harari LH , Baum M , Strous RD . Postpartum anxiety in a cohort of women from the general population: risk factors and association with depression during last week of pregnancy, postpartum depression and postpartum PTSD. Isr J Psychiatry Relat Sci. 2014;51(2):128. 25372562

[37] Verreault N , Da Costa D , Marchand A , Ireland K , Banack H , Dritsa M , et al. PTSD following childbirth: a prospective study of incidence and risk factors in Canadian women. J Psychosom Res. 2012;73(4):257–63. 22980529 10.1016/j.jpsychores.2012.07.010

[38] Vigod SN , Villegas L , Dennis CL , Ross LE . Prevalence and risk factors for postpartum depression among women with preterm and low-birth-weight infants: a systematic review. BJOG Int J Obstet Gynaecol. 2010;117(5):540–50. 10.1111/j.1471-0528.2009.02493.x 20121831

[39] Lebel C , MacKinnon A , Bagshawe M , Tomfohr-Madsen L , Giesbrecht G . Elevated depression and anxiety symptoms among pregnant individuals during the COVID-19 pandemic. J Affect Disord. 2020;277:5–13. 32777604 10.1016/j.jad.2020.07.126 PMC7395614

[40] Berthelot N , Lemieux R , Garon-Bissonnette J , Drouin-Maziade C , Martel É , Maziade M . Uptrend in distress and psychiatric symptomatology in pregnant women during the coronavirus disease 2019 pandemic. Acta Obstet Gynecol Scand. 2020;99(7):848–55. 32449178 10.1111/aogs.13925

[41] Matsushima M , Horiguchi H . The COVID-19 pandemic and mental well-being of pregnant women in Japan: need for economic and social policy interventions. Disaster Med Public Health Prep. 2020;10:1–6. 10.1017/dmp.2020.334 PMC7642494 32907687

[42] Feldman R , Granat AD , Pariente C , Kanety H , Kuint J , Gilboa-Schechtman E . Maternal depression and anxiety across the postpartum year and infant social engagement, fear regulation, and stress reactivity. J Am Acad Child Adolesc Psychiatry. 2009;48(9):919–27. 19625979 10.1097/CHI.0b013e3181b21651

[43] Field T . Postpartum depression effects on early interactions, parenting, and safety practices: a review. Infant Behav Dev. 2010;33(1):1–6. 19962196 10.1016/j.infbeh.2009.10.005 PMC2819576

