# Supplementary material for: The use of personal protective equipment as an independent factor for developing depressive and post-traumatic stress symptoms in the postpartum period
Source: Eur Psychiatry. 2021 May 4;64(1):e34. doi: 10.1192/j.eurpsy.2021.29 (PMC8260566; doi:10.1192/j.eurpsy.2021.29)
Supplement: Supplementary file 1 [file S0924933821000298sup001.docx]

Hello,

In the following survey you will find questions regarding your current birth experience and your postpartum status. The questionnaire is important in order for us to understand the consequences of childbirth and postpartum in times of the Coronavirus.
The participation in this research is voluntary. You may stop the survey at any given moment. The privacy of your answers is guaranteed, and publications of study results will be performed anonymously with no personal details of participants.

The filling out of this survey will be considered as an informed consent for participating in this research.

Thank you in advance for your cooperation!

1. Please fill in the 4 last digits of your ID number ___________

**DEMOGRAPHICS**

1. Age
2. Gender (woman, other)
3. Country of birth (Israel, other)
4. Religion (Judaism, Islam, Christianity, not religious, other)
5. Religious level (secular, traditional, orthodox, ultra-orthodox, other)
6. Marital status (single, in an unmarried relationship, married, divorced, separated, widowed, other)
7. Educational level (primary, high school graduate, first degree, second degree, third degree and up)
8. Average household income (the current average household income in Israel is 19,500 NIS) (much below average, below average, average, above average, much above average)
9. Do you have any physical, cognitive or mental (diagnosed or undiagnosed) disability, such as ADHD, depression, autism, deafness, chronic medical conditions? (yes, no), if yes- what is your disability?

**PERSONAL PROTECTIVE EQUIPMENT**

1. Did the medical staff wear personal protective equipment during your delivery? Please mark which items they were wearing (mask, protective glasses, robe, gloves) how difficult was this for you? (not at all, a little bit difficult, fairly difficult, very difficult, extremely difficult)

**FEAR OF COVID-19**

How much do you agree with the following statements?

|  | Definitely disagree | Disagree | Does not agree and does not deny | Agree | Definitely agree |
| --- | --- | --- | --- | --- | --- |
| 1. I am most afraid of COVID-19 |  |  |  |  |  |
| 1. It makes me uncomfortable to think about COVID-19 |  |  |  |  |  |
| 1. My hands become clammy when I think about COVID-19 |  |  |  |  |  |
| 1. I am afraid of losing my life because of COVID-19 |  |  |  |  |  |
| 1. When watching news and stories about COVID-19 on social media, I become nervous or anxious |  |  |  |  |  |
| 1. I cannot sleep because I’m worrying about getting COVID-19 |  |  |  |  |  |
| 1. My heart races or palpitates when I think about getting COVID-19 |  |  |  |  |  |

**CITY BIRTH TRAUMA SCALE**

This questionnaire asks about your experience during the birth of your most recent baby. It asks about potential traumatic events during (or immediately after) the labour and birth, and whether you are experiencing symptoms that are reported by some women after birth. Please tick the responses closest to your experience.

| During the labour, birth and immediately afterwards: | Score 1 | Score 0 |
| --- | --- | --- |
| Q1. Did you believe you or your baby would be seriously injured? | Yes | No |
| Q2. Did you believe you or your baby would die? | Yes | No |

The next questions ask about symptoms you may have experienced. Please indicate how often you have experienced the following symptoms in the last week:

| Symptoms about the birth* | Not at all | Once | 2 - 4 times | 5 or more times |
| --- | --- | --- | --- | --- |
| Q3. Recurrent unwanted memories of the birth (or parts of the birth) that you can’t control | 0 | 1 | 2 | 3 |
| Q4. Bad dreams or nightmares about the birth (or related to the birth) | 0 | 1 | 2 | 3 |
| Q5. Flashbacks to the birth and/or reliving the experience | 0 | 1 | 2 | 3 |
| Q6. Getting upset when reminded of the birth | 0 | 1 | 2 | 3 |
| Q7. Feeling tense or anxious when reminded of the birth | 0 | 1 | 2 | 3 |
| Q8. Trying to avoid thinking about the birth | 0 | 1 | 2 | 3 |
| Q9. Trying to avoid things that remind me of the birth (e.g. people, places, TV programs) | 0 | 1 | 2 | 3 |
| Q10. Not able to remember details of the birth | 0 | 1 | 2 | 3 |
| Q11. Blaming myself or others for what happened during the birth | 0 | 1 | 2 | 3 |
| Q12. Feeling strong negative emotions about the birth (e.g. fear, anger, shame) | 0 | 1 | 2 | 3 |

* Although these questions refer to the birth, many women have symptoms about events that happened just before or after birth. If this is the case for you, and the events were related to pregnancy, birth or the baby then please answer for these events.

| Symptoms that began or got worse since the birth | Not at all | Once | 2 - 4 times | 5 or more times |
| --- | --- | --- | --- | --- |
| Q13. Feeling negative about myself or thinking something awful will happen | 0 | 1 | 2 | 3 |
| Q14. Lost interest in activities that were important to me | 0 | 1 | 2 | 3 |
| Q15. Feeling detached from other people | 0 | 1 | 2 | 3 |
| Q16. Not able to feel positive emotions (e.g. happy, excited) | 0 | 1 | 2 | 3 |
| Q17. Feeling irritable or aggressive | 0 | 1 | 2 | 3 |
| Q18. Feeling self-destructive or acting recklessly | 0 | 1 | 2 | 3 |
| Q19. Feeling tense and on edge | 0 | 1 | 2 | 3 |
| Q20. Feeling jumpy or easily startled | 0 | 1 | 2 | 3 |
| Q21. Problems concentrating | 0 | 1 | 2 | 3 |
| Q22. Not sleeping well because of things that are not due to the baby’s sleep pattern | 0 | 1 | 2 | 3 |
| Q23. Feeling detached or as if you are in a dream | 0 | 1 | 2 | 3 |
| Q24. Feeling things are distorted or not real | 0 | 1 | 2 | 3 |

If you have any of these symptoms:

| Q25. When did these symptoms start? | |
| --- | --- |
| Before the birth | 0 |
| In the first 6 months after birth | 1 |
| More than 6 months after birth | 2 |
| Not applicable (I have no symptoms) |  |

| Q26. How long have these symptoms lasted? | |
| --- | --- |
| Less than 1 month | 0 |
| 1 to 3 months | 1 |
| 3 months or more | 2 |
| Not applicable (I have no symptoms) |  |

| Q27. Do these symptoms cause you a lot of distress? | Yes 2 | No 0 | Sometimes 1 |
| --- | --- | --- | --- |
| Q28. Do they prevent you doing things you usually do (e.g. socialising, daily activities)? | Yes 2 | No 0 | Sometimes 1 |
| Q29. Could any of these symptoms be due to medication, alcohol, drugs, or physical illness? | Yes 2 | No 0 | Maybe 1 |

**EDINBURGH POSTNATAL DEPRESSION SCALE (EPDS)**

Please enclose in each group the sentence that best describes your feelings in the past month, including today.

In the last past month:

1. I have been able to laugh and see the funny side of things
   1. As much as I always could
   2. Not quite as much now
   3. Definitely not so much now
   4. Not at all
2. I have looked forward with enjoyment to things
   1. As much as I ever did
   2. Rather less than I used to
   3. Definitely less than I used to
   4. Hardly at all
3. I have blamed myself unnecessarily when things  went wrong
   1. Yes, most of the time
   2. Yes, some of the time
   3. Not very often
   4. No, never
4. I have been anxious or worried for no good reason
   1. No, not at all
   2. Hardly ever
   3. Yes, sometimes
   4. Yes, very often
5. I have felt scared or panicky for no very good reason
   1. Yes, quite a lot
   2. Yes, sometimes
   3. No, not much
   4. No, not at all
6. Things have been getting on top of me
   1. Yes, most of the time I haven't been able to cope at all.
   2. Yes, sometimes I haven't been coping as well as usual
   3. No, most of the time I have coped quite well.
   4. No, I have been coping as well as ever.
7. I have been so unhappy that I have had difficulty sleeping
   1. Yes, most of the time
   2. Yes, sometimes
   3. Not very often
   4. No, not at all
8. I have felt sad or miserable
   1. Yes, most of the time
   2. Yes, quite often
   3. Not very often
   4. No, not at all
9. I have been so unhappy that I have been crying
   1. Yes, most of the time
   2. Yes, quite often
   3. Only occasionally
   4. No, never
10. The thought of harming myself has occurred to me
    1. Yes, quite often
    2. Sometimes
    3. Hardly ever
    4. Never
